# Treatment decision satisfaction and regret after focal HIFU for localized prostate cancer

**DOI:** 10.1007/s00345-020-03301-0

**Published:** 2020-06-12

**Authors:** Niklas Westhoff, Ramona Ernst, Karl Friedrich Kowalewski, Laura Schmidt, Thomas Stefan Worst, Maurice Stephan Michel, Jost von Hardenberg

**Affiliations:** 1 Department of Urology and Urosurgery, Medical Faculty Mannheim, Heidelberg University, Theodor-Kutzer-Ufer 1–3, 68167 Mannheim, Germany; 2 Department of Health Psychology, Institute of Psychology, Heidelberg University, Hauptstraße 47-51, 69117 Heidelberg, Germany

**Keywords:** Focal therapy, High-intensity focused ultrasound, Prostate neoplasms, Recurrence, Regret

## Abstract

**Purpose:**

Focal therapies (FTs) are investigated within prospective studies on selected patients treated for localized prostate cancer (PCa). Benefits are preservation of genitourinary function and reduced complications, but follow-up is elaborate and is associated with uncertainty as cancer-free survival appears to be lower compared to standard radical treatments. The aim of this study was to analyse patient-reported acceptance of FT and evaluate factors associated with treatment decision regret.

**Methods:**

52 patients who received focal high-intensity focused ultrasound for low- to intermediate-risk PCa between 2014 and 2019 within two prospective trials were eligible for a survey regarding PCa-related treatment regret and quality-of-life (Clark’s scale) and the following potential predictors: sociodemographic variables, Charlson Comorbidity Index, subjective aging (AARC-10 SF), and general health-related quality-of-life (SF-12). Cancer persistence/recurrence (multiparametric MRI and fusion biopsy after 12 months) and functional outcomes (EPIC-26 UI/UIO/S) data were also included in this study.

**Results:**

The overall survey response rate was 92.3% (48/52 patients). Median follow-up was 38 months (interquartile range = 25–50 months). In total, ten patients (20.8%) reported treatment decision regret. In univariable analyses, a clinically meaningful increase in urinary incontinence showed a significant association (OR 4.43; 95% CI 0.99–20.53; *p* = 0.049) with regret. Cancer recurrence (OR 12.31; 95% CI 1.78–159.26; *p = 0.023*) and general health worry as a domain of Clark’s scale (OR 1.07; 95% CI 1.03–1.14; *p* < 0.01) were predictors of regret in a multivariable logistic regression model (AUC = 0.892).

**Conclusion:**

Acceptance of FT is comparable to standard treatments. Extensive follow-up including regular PSA testing does not cause additional regret but careful patient selection and information before FT is crucial.

**Electronic supplementary material:**

The online version of this article (10.1007/s00345-020-03301-0) contains supplementary material, which is available to authorized users.

## Introduction

Prostate cancer (PCa) is the second most frequent cancer in men and the fifth leading cause of death from cancer worldwide [1]. In patients with a life expectancy of more than 10 years, cancers at a low risk for progression and metastasis can be monitored with Active Surveillance (AS) protocols to spare or postpone radical treatment. If a clinically significant localized PCa is present or if patients ask for active treatment, curative treatment options comprise surgery [robotic assisted or open radical prostatectomy (RP)] and radiation therapy (external beam radiation therapy (EBRT) and/or brachytherapy (BT)) [2].

A patient’s decision for a particular therapy option in localized PCa is mainly based on the spectrum of side-effects since PCa-specific mortality is low irrespective of the kind of treatment [3]. Side-effects after radiation therapy include voiding symptoms, bowel dysfunction, sexual dysfunction, and secondary malignancies. After RP, urinary incontinence and sexual dysfunction are the most relevant side-effects [3, 4]. Focal therapy (FT) has been developed to reduce the side-effects of whole-gland treatments, to maintain genitourinary function, and to provide equivalent oncologically safe treatment. Ablative approaches such as high-intensity focused ultrasound (HIFU), irreversible electroporation, and focal BT treat intraprostatic cancer lesions locally while sparing benign organ tissue [5]. Although the overall quality of evidence of the oncological effectiveness compared to standard treatments is still low and there is a lack of long-term follow-up, recent data show a favourable toxicity profile for FT [5].

Decision-making is a sophisticated process for patients: an unfavourable outcome, either regarding the oncological prognosis or quality-of-life, can lead to regret over the treatment selected, the choice itself, or the outcome [6]. During recent years, measurement of regret after PCa treatment has become an emerging instrument to define factors associated with acceptance of therapy choice [7–10].

To implement FTs into the algorithm of PCa treatment, it is first necessary to characterize how patients evaluate their decision for and experience with this treatment. However, no data exists on the postoperative acceptance of FT, patients´ well-being, and self-reported treatment outcomes. Thus, the aim of this prospective cohort study was to determine regret of FT and to identify potentially associated oncologic, sociodemographic, functional, age-related, and quality-of-life factors as well as the influence of informed decision-making.

## Subjects and methods

### Patients

Between October 2014 and July 2019, 61 patients were treated by HIFU within the registered prospective single-center trials FOXPRO and FOXPRO-REGISTER (at germanctr.de; DRKS00007105 and DRKS00009021). The studies were approved by the institutional ethical review committee (No. 2014-423 M-MA and 2015-401 M-MA). All patients gave written informed consent. Inclusion criteria were a low-to-intermediate-risk PCa and a PI-RADS 3–5 target lesion as determined by multiparametric magnetic resonance imaging (mpMRI) of the prostate. If the mpMRI was negative, a biopsy-proven cancer limited to one prostate zone was accepted. The technique of HIFU and the study workflow were described in detail previously [11].

### Data acquisition and survey questionnaire

By study protocol, oncological treatment outcomes and patient-reported functional outcomes were recorded at 3, 6, 12, 24, and 36 months. Oncological follow-up included repeated measurements of prostate-specific antigen (PSA) levels. At 12 months, or before in cases of rising PSA, patients received a control mpMRI and an MRI/ultrasound-fusion prostate biopsy to assess recurrence or persistence of cancer. The genitourinary functional outcome was measured by validated questionnaires by comparing values before HIFU treatment (baseline) and at follow-up. We used the well-established Expanded Prostate Cancer Index Composite 26 (EPIC-26) questionnaire set, which is a patient-reported outcome questionnaire to monitor health-related quality-of-life outcomes among patients after PCa therapy. The three domains for urinary incontinence (EPIC-26 UI), irritative/obstructive symptoms (EPIC-26 UIO), and sexuality (EPIC-26 S) were selected (Online Resource 1). The summary scores for the domains ranged from 0 to 100 with lower values representing worsening genitourinary function [12]. Established Minimal Important Differences (MIDs) were used to determine the number of patients who reported a clinically meaningful change of outcome during follow-up [13].

The survey data for regret of therapy were collected between September and October of 2019. Patients received a questionnaire that was subdivided into different parts. Part one requested sociodemographic parameters, which were patient age, marital status, housing situation, education, employment status, and health insurance. Part two consisted of questions regarding chronic and oncological diseases for calculation of the Charlson Comorbidity Index. This score weights existing comorbidities to predict a 10-year survival probability [14]. For part three, Clark’s validated scale was used to assess PCa-related quality-of-life. Nineteen statements are allocated to the 5 domains “treatment decision regret” (five statements), “having made an informed decision” (four statements), “health worry” (six statements), “PSA concern” (two statements), and “outlook” (two statements). Ratings of how true the statements were perceived ranged from 1 (not at all) to 5 (very much) and scales were scored afterwards from 0 to 100 according to Clark’s recommended scoring procedure. A score of ≥ 40 was classified as a treatment decision regret [9] (Online Resource 2). In part four of the survey, patients’ self-perceptions of aging were assessed using the Awareness of Age-Related Change Short Form (AARC-10 SF). The short form was developed in 2017 from the 50 item AARC and contains five statements that describe gains of aging and five statements that describe losses (e.g.,”With my increasing age, I realize that … I have a better sense of what is important to me” or ”… I feel more dependent on the help of others”). Ratings of the statements ranged from 1 (not at all) to 5 (very much) and were summed up as two separate scores where higher values indicate higher levels of perceived gains or losses. In addition, we assessed the “subjective” or “felt” age (“Many people feel older or younger than they actually are. Fill in the age (in years) that you feel most of the time:…”) [15]. Following the recommendation of Rubin and Berntsen, we calculated the proportional discrepancy (discrepancy between felt and chronological age divided by chronological age) [16]. Part five was the Short Form Health Survey (SF-12), a shortened version of the SF-36, which is frequently used to collect data on general health-related quality-of-life [17]. A general health rating was assessed by one introductory question which was subsequently used for further association analyses.

### Statistical analyses

Categorical data are provided as absolute and relative frequencies. Location parameters of continuous data are reported as median or mean. Measures of variation are reported as range, interquartile range (IQR), or standard deviation (SD). For sociodemographic, histopathological, functional, quality-of-life, and age-related variables, the association with regret of FT was evaluated by univariable analyses. Factors that predict regret of FT were defined by a multivariable logistic regression model which included statistically significant variables of univariable analyses. Receiver operating characteristic (ROC) curves were generated for the independent predictors of regret. Statistical significance was set at *p* values < 0.05. Statistical analyses were performed using R version 3.5.2 (R project, R Foundation for Statistical Computing) and JMP^®^ (version 14.0.0, SAS Institute Inc., USA).

## Results

Of the 61 patients who received HIFU therapy, 52 patients were eligible for the survey. Reasons for exclusion were: seven patients discontinued study follow-up; 1 patient had developed dementia; and 1 patient died (a non-PCa-related event). A total of 48 patients completed questionnaires, corresponding to a survey response rate of 92.3%. All the four patients who did not respond showed residual or recurrent cancer in the control biopsy after HIFU treatment. The mean patient age was 68.0 years (SD 7.9 years) at HIFU therapy and 71.0 years (SD 8.2 years) at survey. Whereas the median follow-up time (from HIFU to survey completion) was 38 months (range of 25–50 months), in five patients (10.4%), the interval was less than 12 months. These patients had not yet received the control mpMRI and biopsy. The sociodemographic baseline characteristics of the cohort are shown in Table 1. Most patients were married, living with a partner or flatmate, and in retirement.

**Table 1:** Sociodemographic characteristics

Parameter	Value
Number of patients with completed regret of therapy follow-up	48
Mean patient age, years (SD)	
At HIFU	68.0 (7.9)
At survey	71.0 (8.2)
Median time from HIFU to survey, months (IQR)	38 (25–50)
Time from HIFU to survey < 12 months (*n*, %)	5 (10.4)
Marital status (*n*, %)	
Yes	40 (83.3)
No	5 (10.4)
n/a	3 (6.3)
Housing situation (*n*, %)	
With partner/shared flat	41 (85.4)
Alone	4 (8.3)
n/a	3 (6.3)
Educational qualification (*n*, %)	
University/college	26 (54.2)
Lower	21 (43.8)
n/a	1 (2.1)
Employment status (*n*, %)	
Full- or part-time	10 (20.8)
Retired	37 (77.1)
n/a	1 (4.3)
Insurance (*n*, %)	
Private	18 (37.5)
Statutory or combined private	28 (58.4)
n/a	2 (4.2)

*HIFU* high-intensity focused ultrasound, *n/a* not applicable

Results of the control biopsy were available for 70.8% of the patients. Beside the five patients (10.4%) with an outstanding MRI and biopsy, nine patients (18.8%) rejected the biopsy due to decreasing PSA levels or unsuspicious mpMRI. Cancer was found in the control biopsies of 22 patients (45.8%), of whom 12 patients (25%) had a Gleason Score of ≥ 3 + 4 (clinically significant cancer). One-third of patients (*n* = 16) received a salvage therapy by radical prostatectomy (*n* = 7), EBRT (*n* = 6), re-HIFU (*n* = 2), or androgen deprivation therapy (*n* = 1). A clinically meaningful impairment of urinary continence was recorded in 12 patients (25%), of irritative and obstructive symptoms in 11 patients (22.9%), and of erectile function in 14 patients (29.2%) **(**Table 2).

**Table 2:** Comparison of oncological and functional outcomes prior to high-intensity focused ultrasound (HIFU) and at time of follow-up 12 months after HIFU

Parameter	At baseline (prior to HIFU)	At follow-up (after HIFU)
Prostate biopsy (*n*, %)		
Completed	48 (100)	34 (70.8)
Rejected by patient		9 (18.8)
Outstanding		5 (10.4)
PSA, ng/ml		
Increase (*n*, %)		16 (33.3)
No increase (*n*, %)		32 (66.6)
Gleason score at biopsy (*n*, %)		
3 + 3	26 (54.2)	10 (20.8)
3 + 4	17 (35.4)	8 (16.7)
≥ 4 + 3	5 (10.4)	4 (8.3)
Salvage therapy (*n*, %)		
Yes		16 (33.3)
No		32 (66.6)
Voiding function		
EPIC-26 Urinary Incontinence domain		
Mean (range)	95.6 (66.8–100)	89.2 (46–100)
Clinically meaningful impairment (*n*, %) ^a^		12 (25)
n/a		10 (20.8)
EPIC-26 Urinary irritative/obstructive domain		
Mean (range)	90.1 (37.5–100)	90.3 (56.3–100)
Clinically meaningful impairment (*n*, %) ^b^		11 (22.9)
n/a		11 (22.9)
Erectile function		
EPIC-26 Sexual domain		
Mean (range)	68.3 (0–100)	58.2 (8.3–100)
Clinically meaningful impairment (*n*, %) ^c^		14 (29.2)
n/a		10 (20.8)

*HIFU* high-intensity focused ultrasound, *EPIC-26* expanded prostate cancer index composite 26, *n/a* not applicable

^a^Minimal important difference (MID): a decrease of ≥ 6 points from baseline to latest available follow-up

^b^MID of ≥ 5 points

^c^MID of ≥ 10 points

Most men reported being in good-to-excellent general health (85.4%) (SF-12). Of five patients who reported poor or very poor health, three received salvage therapy due to cancer detection in the control biopsy. Patients usually felt younger than their chronological age, which resulted in a mean discrepancy of − 10.4 years (SD 9.8 years) (AARC-10 SF). This corresponded to a proportional discrepancy score of − 0.14 (SD 0.12). There was a higher likelihood to perceive gains rather than losses in terms of the variance of patients’ age-related self-perceptions (mean score 16.7 vs. 11.0) (Table 3).

**Table 3:** General health status, comorbidity, awareness of age-related change and prostate cancer-related quality-of-life of patients at follow-up after high-intensity focused ultrasound (HIFU)

Parameter	Value
SF-12 health-related QoL	
General health rating	
Excellent/very good, (*n*, %)	13 (27.1)
Good (*n*, %)	28 (58.3)
Poor/very poor (*n*, %)	5 (10.4)
Physical component score (mean, SD)	48.3 (6.9)
Mental component score (mean, SD)	53.1 (7.3)
No. of patients with physical component score > 50 (%)	22 (45.8)
No. of patients with mental component score > 50 (%)	36 (75)
Item missing (*n*, %)	2 (4.2)
Charlson Comorbidity Index, % (mean, SD)	29.8 (25.3)
Measures of subjective age (mean, SD)	
AARC-10 SF gains^a^	16.7 (3.3)
AARC-10 SF losses^a^	11.0 (3.3)
Discrepancy of felt and chronological age in years	− 10.4 (9.8)
Proportional discrepancy score^b^	− 0.14 (0.12)
Item missing (*n*, %)	3 (6.3)
Clark’s prostate cancer-related QoL	
Health worry (mean, SD)^c^	42.3 (22.6)
PSA concern (mean, SD)^d^	88.0 (15.1)
Outlook (mean, SD)^e^	51.6 (23.2)
Informed decision index (mean, SD)^f^	88.7 (17.1)
Treatment regret index ≥ 40 (*n*, %)^g^	10 (20.8)
Item missing (*n*, %)	1 (2.1)

*AARC-10 SF* awareness of age-related change short form, *HIFU* high-intensity focused ultrasound, *SF-12* short form health survey, *QoL* quality of life

^a^Higher values indicate higher levels of perceived gains or losses

^b^Higher values indicate less discrepancy of felt and chronological age

^c^Higher values indicate greater worry

^d^Higher values indicate greater concern

^e^Higher values indicate better outlook

^f^Higher values indicate more informed decision

^g^Values ≥ 40 indicate regret

According to Clark’s PCa-related quality-of-life assessment, ten patients (20.8%) described treatment decision regret. Comparing patients with regret to those without, 80% vs. 36.8% (*p* = 0.025), respectively, had cancer at control biopsy. More patients reported a clinically meaningful worsening of incontinence (50% vs. 18.4%; *p* = 0.049) and levels of health worry were higher (41.67 vs. 25.0; *p* = 0.003). Table 4 demonstrates the results of uni- and multivariable analyses of factors associated with regret of therapy. In the multivariable logistic regression model, cancer at control biopsy and general health worry were predictors of treatment decision regret (*p* = 0.025 and *p* = 0.009, respectively). Combining both variables results in an AUC of 0.892 (Online Resource 3).

**Table 4:** Logistic regression model of factors associated with regret of focal therapy

Variable	Direction and unit	Univariable analyses			Multivariable analyses		
		Odds Ratio	95% CI	*p* value	Odds Ratio	95% CI	*p* value
Age ≥ 65 years	Yes vs. no	1.89	0.45–9.80	0.4047			
Married	Yes vs. no	1.00	0.12–20.99	1.0000			
Housing situation	Living with partner or shared flat (vs. living alone)	0.73	0.08–15.78	0.7941			
Education	University or college (vs. lower)	0.58	0.13–2.53	0.4684			
Employment	Full-/part time (vs. retired)	0.40	0.02–2.66	0.4198			
Insurance	Private/combined (vs. statutory)	0.53	0.10–2.32	0.4125			
PSA increase	Yes vs. no	1.44	0.32–6.05	0.6163			
Cancer recurrence	Yes vs. no	6.86	1.47–49.89	**0.0250**	12.31	1.78–159.26	**0.0225**
Recurrent Gleason Score 3 + 3	Yes vs. no	1.33	0.23–7.97	0.7464			
Recurrent Gleason Score ≥ 3 + 4	Yes vs. no	0.75	0.13–4.40	0.7464			
Salvage therapy	Yes vs. no	2.45	0.58–10.57	0.2165			
Urinary incontinence	CMI vs. no CMI	4.43	0.99–20.53	**0.0497**	5.67	0.76–56.97	0.1010
Urinary irritative/obstructive symptoms	CMI vs. no CMI	1.61	0.30–7.37	0.5514			
Erectile dysfunction	CMI vs. no CMI	0.54	0.07–2.58	0.4780			
General health rating	Excellent/very good/good (vs. poor/very poor)	0.26	0.05–3.10	0.3090			
Charlson Comorbidity Index	Increasing units	0.11	0.003–2.13	0.1783			
Proportional age discrepancy score	Increasing units	5.55	0.01–5922.09	0.5997			
AARC-10 gains	Increasing units	1.01	0.82–1.27	0.8968			
AARC-10 losses	Increasing units	0.99	0.79–1.22	0.8961			
Health worry	Increasing units	1.06	1.03–1.12	**0.0034**	1.07	1.03–1.14	**0.0096**
PSA concern	Increasing units	1.02	0.97–1.09	0.4521			
Outlook	Increasing units	0.99	0.96–1.02	0.5354			
Informed decision making	Increasing units	0.96	0.91–1.00	0.0987			

Bold values are indicate statistically significant variables

Urinary incontinence, urinary irritative/obstructive symptoms, erectile dysfunction: domains of EPIC-26; General health rating: Item of SF-12; Health worry, PSA concern, Outlook, Informed decision making: domains of Clark’s scale

*AARC-10* awareness of age-related change short form, *CI* confidence interval, *CMI* clinically meaningful impairment, *PSA* prostate specific antigen

## Discussion

Treatment decision in localized PCa challenges patients and is, besides the goal of curing cancer, mainly based on therapy-related side-effects and patients’ preferences [18, 19]. Consequently, patient-reported outcomes should be gathered and presented in a comprehensible manner. Patients can better choose a treatment by assessing potential physical functional outcomes like sexual dysfunction and incontinence, and perceived psychosocial consequences such as loss of libido and depression. Therefore, measuring treatment decision regret is of increasing interest in localized PCa, because it quantifies how patients rate their treatment [7, 9]. A growing body of knowledge in this field can guide urologists to improve patient information and shared decision-making.

In this analysis of patients treated by focal HIFU, 20.8% of patients (10/48) reported treatment decision regret. It is relevant to note, that these results are generated from study patients. Since focal PCa therapies are not standardised and different approaches and technologies exist, it is critical to perform prospective trials when evaluating them [20]. These study protocols include follow-ups to measure PSA at defined intervals and another mpMRI and prostate biopsy for control after treatment. In cases of cancer persistence or recurrence, salvage therapies might be considered. Some low-risk cancers may undergo AS. In addition, adverse events, functional status, and quality-of-life should be measured during follow-up [20]. Although undoubtedly indicated, these frequent follow-up visits can lead to distress and anxiety and consequently increase the perception of decision regret.

The extent of decision regret for FT is comparable with regret after standard treatments of localized PCa. In a recent prospective multicenter study, 23% of patients reported a clinically relevant treatment regret after 12 months, irrespective of the kind of treatment. Extent of regret was 37% after EBRT, 23% after RP, 20% after AS, and 18% after BT, but there was no statistical significant difference (*p* > 0.05) [21]. Hoffman et al. evaluated regret among long-term survivors 15 years after local therapy (*n* = 934). The overall regret rate was 14.6%, with 16.6% expressing regret after EBRT/BT, 15% after RP, and 8.2% after conservative management [8]. In a systematic review of 28 articles, regret rates varied between 0.5 and 31% for RP, 9.2–24% for EBRT, and 0–24% for BT [7]. Although FTs are new and knowledge of potential complications, early and late side-effects, and oncological outcomes are still very limited, patients who received FT did not express more regret about their treatment decision compared to patients who chose a radical treatment. At the same time, a potential preservation of genitourinary function did not improve regret rates in favour of FT. Notably, within these studies, regret was also measured by instruments other than the Clark’s scale (e.g., Decision Regret Scale [22]), thereby reducing comparability. Moreover, there are no scales that classify whether patients feel lower or higher levels of regret making it difficult to compare the extent of regret between different cohorts.

Two situations are responsible for development of regret after treatment when there is no definitive preferable clinical therapy (i.e., a “preference-sensitive” cancer-related decision): either a lack of positive outcomes or occurrence of worse outcomes [8]. In our analyses, cancer persistence/recurrence at control biopsy was strongly correlated with treatment decision regret. Thus, these patients experienced a lack of an expected positive oncological outcome. As part of our study protocol and according to recommendations, we performed a detailed therapy control by mpMRI and a targeted biopsy of the treated and a systematic biopsy of the untreated prostate tissue. The overall cancer recurrence rate in this study was 45.8%, including clinically significant and insignificant cancers. Recent FT trial data show similar rates of treatment failures among different modalities [23, 24]. Major reasons for these high rates are the multifocality of PCa, imaging and biopsy errors, and inappropriate patient selection [25]. Consequently, patients should be well informed about the risk of relapse to reduce regret after treatment. Only those patients who accept potential further salvage treatments should be admitted to FT. These patients should also be informed that the evidence of outcome from salvage treatments is very low but that preliminary data indicate slightly worse oncological and functional outcomes [26]. Interestingly, patients with a low-risk PCa at control biopsy, defined by a Gleason Score of 3 + 3, were more likely to express regret than patients with clinically significant cancers. In addition, receiving a salvage therapy showed no correlation in this series. One explanation may be that patients who received salvage therapies were satisfied with its course and outcome. Patients with low-risk cancers frequently (60%) underwent AS which could have caused emotional distress and a feeling of loss of control over their cancer, or otherwise led to decreased satisfaction when AS had already been a primary option before choice of FT.

Regarding functional outcomes, 25% of patients reported a clinically meaningful impairment of urinary continence at their last follow-up which was associated with regret in univariable analyses but not in the multivariable model. Urinary irritative/obstructive symptoms and erectile dysfunction did not predict regret. A significant correlation with urinary incontinence was also reported in a comprehensive review by Christie et al. [7]. Although urinary incontinence is more frequent after RP and EBRT, it can increase after FT and especially for apical cancers. If patients were previously unprepared, unexpected urinary incontinence could cause a higher level of disappointment [27, 28].

A greater general health worry was another significant predictor of decision regret. It is reasonable that patients who are highly concerned about their health status, results of medical examinations, or disease-related death are sensitive to worse outcomes [9]. These patients should be identified before decision-making, for example by questionnaires, to optimally provide informational content.

The role of PSA testing for follow-up after treatment is under debate since there is no nadir due to tissue preservation. This may cause uncertainty for patients. However, a PSA increase or general PSA concern did not affect acceptance of FT. Moreover, in this cohort, social circumstances (marital and housing situation, employment and insurance status) and educational level did not influence occurrence of regret. In contrast, van Stam et al. showed a correlation with educational level in standard treatments [21]. Although patients of higher age (≥ 65 years) were more likely to express regret and patients generally felt younger than their true age, there was no significant correlation between regret and age itself, felt age discrepancy, and perceived age-related gains or losses.

Our study has some limitations. First, our sample size was small which limits statistical significance. However, our sample size was similar to other prospective studies of FT and is explained by its novelty and highly selected patient population. Second, it is questionable whether these results after focal HIFU therapy can be transferred to other FT modalities. Different recurrence rates and side-effects might affect regret rates. Nevertheless, HIFU is one of the most common FTs and, therefore, more data for this treatment alternative are highly needed. Third, the time interval between FT and assessment of regret varied between the patients and was longer for those reporting regret. Reports from long-term follow-up after standard treatments also showed that regret increased over time [8]. Last, we cannot make a direct comparison to standard treatment options. Instruments that measure regret vary, but the Clark’s scale that was used in this study is one of the two most common tools.

In conclusion, in localized PCa “preference-sensible” treatment decisions are common. Analysing regret of treatment decisions is a helpful instrument to measure acceptance after FT. This first prospective cohort study of patients treated by FT showed that overall treatment acceptance was comparable to standard treatments. Cancer recurrence, functional outcomes and a general health worry can lead to treatment decision regret but follow-up by regular PSA testing with its particular uncertainty in interpretation after FT does not. Future studies might address moderating variables related to health worry with the potential to explain variance in decision regret or explore therapy-related expectations within larger patient populations. This will pave the way for comparative studies analysing regret of FT versus standard treatment options. To circumvent treatment decision regret, awareness of current cancer persistence/recurrence rates and side-effects will allow urologists to provide comprehensive information and further improve careful patient selection and shared decision-making.

## Electronic supplementary material

Below is the link to the electronic supplementary material.

Supplementary file1 (PDF 74 kb)

Supplementary file2 (PDF 57 kb)

Supplementary file3 (PDF 37 kb)

## Data Availability

The data that support the findings of this study are available from the corresponding author upon reasonable request.
