# Supplementary material for: Treatment decision satisfaction and regret after focal HIFU for localized prostate cancer
Source: World J Urol. 2020 Jun 12;39(4):1121–9. doi: 10.1007/s00345-020-03301-0 (PMC8124049; doi:10.1007/s00345-020-03301-0)
Supplement: Supplementary file 1 — Supplementary file1 (PDF 74 kb) [file 345_2020_3301_MOESM1_ESM.pdf]

Title: Treatment decision satisfaction and regret after focal HIFU for localized prostate cancer

Journal: World Journal of Urology

Authors: Niklas Westhoff, Ramona Ernst, Karl Friedrich Kowalewski, Laura Schmidt, Thomas Stefan Worst, Maurice-Stephan Michel, Jost von Hardenberg

Corresponding author:

Niklas Westhoff, M.D.

Department of Urology and Urosurgery, Medical Faculty Mannheim, Heidelberg University, Mannheim, Germany

Theodor-Kutzer-Ufer 1 - 3

68167 Mannheim, Germany

Fon: +49 621 383 8331

Fax: +49 621 383 2184

Email: niklas.westhoff@medma.uni-heidelberg.de

Expanded Prostate Cancer Index Composite
Wei et al., Urology. 2000. 56(6):899-905

EPIC

The Expanded Prostate Cancer Index Composite

This questionnaire is designed to measure Quality of Life issues in patients with Prostate cancer. To help us get the most accurate measurement, it is important that you answer all questions honestly and completely.

Remember, as with all medical records, information contained within this survey will remain strictly confidential.

Today`s Date (please enter date when survey completed): Month______Day_____Year_____

Name (optional):____

Date of Birth (optional): Month______Day_____Year_____

URINARY FUNCTION
This section is about your urinary habits. Please consider **ONLY THE LAST 4 WEEKS**.

1. Over the **past 4 weeks**, how often have you leaked urine? (Circle one number)

   More than once a day 1
   About once a day.. 2
   More than once a week.. 3
   About once a week.. 4
   Rarely or never.. 5

2. Over the **past 4 weeks**, how often have you urined blood? (Circle one number)

   More than once a day 1
   About once a day.. 2
   More than once a week.. 3
   About once a week.. 4
   Rarely or never.. 5

3. Over the **past 4 weeks**, how often have you had pain or burning with urination? (Circle one number)

   More than once a day 1
   About once a day.. 2
   More than once a week.. 3
   About once a week.. 4
   Rarely or never.. 5

4. Which of the following best describes your urinary control **during the last 4 weeks**? (Circle one number)

   No urinary control whatsoever... 1
   Frequent dribbling.. 2
   Occasional dribbling... 3
   Total control... 4

5. How many pads or adult diapers per day did you usually use to control leakage **during the last 4 weeks**? (Circle one number)

   None.. 0
   1 pad per day... 1
   2 pads per day... 2
   3 or more pads per day.. 3

6. How big a problem, if any, has each of the following been for you **during the last 4 weeks**? (Circle one number on each line)

	No Problem	Very small Problem	Small Problem	Moderate Problem	Big Problem
a. Dripping or leaking urine……………	0	1	2	3	4
b. Pain or burning on urination……….	0	1	2	3	4
c. Bleeding with urination................	0	1	2	3	4
d. Weak urine stream or incomplete emptying………………………………..	0	1	2	3	4
e. Waking up to urinate…………………	0	1	2	3	4
f. Need to urinate frequently during the day………………………………………	0	1	2	3	4

7. Overall, how big a problem has your urinary function been for you **during the last 4 weeks**? (Circle one number)

No problem…………………………………………………………………………….	1
Very small problem………………………………………………………………….	2
Small problem…………………………………………………………………………	3
Moderate problem……………………………………………………………………	4
Big problem…………………………………………………………………………….	5

SEXUAL FUNCTION
The next section is about your current sexual function and sexual satisfaction. Many of the questions are very personal, but they will help us understand the important issues that you face every day. Remember, THIS SURVEY INFORMATION IS COMPLETELY <u>CONFIDENTIAL</u>. Please answer honestly about **THE LAST 4 WEEKS ONLY**.

1. How would you rate each of the following **during the last 4 weeks**? (Circle one number on each line)

	Very poor to none	Poor	Fair	Good	Very good
a. Your level of sexual desire?...............................	1	2	3	4	5
b. Your ability to have an erection?........................	1	2	3	4	5
c. Your ability to reach orgasm (climax)?..............	1	2	3	4	5

2. How would you describe the usual QUALITY of your erections **during the last 4 weeks**? (Circle one number)

None at all..	1
Not firm enough for any sexual activity...	2
Firm enough for masturbation and foreplay only..........................	3
Firm enough for intercourse..	4

3. How would you describe the FREQUENCY of your erections **during the last 4 weeks**? (Circle one number)

I NEVER had an erection when I wanted one..................................	1
I had an erection LESS THAN HALF the time I wanted one............	2
I had an erection ABOUT HALF the time I wanted one...................	3
I had an erection MORE THAN HALF the time I wanted one.........	4
I had an erection WHENEVER I wanted one....................................	5

4. How often have you awakened in the morning or night with an erection **during the last 4 weeks**? (Circle one number)

Never...	1
Less than once a week..	2
About once a week..	3
Several times a week...	4
Daily...	5

5. **During the last 4 weeks**, how often did you have <u>any</u> sexual activity? (Circle one number)

Not at all…………………………………………………………	1
Less than once a week………………………………………	2
About once a week……………………………………………	3
Several times a week…………………………………………	4
Daily…………………………………………………………………	5

6. **During the last 4 weeks**, how often did you have sexual intercourse? (Circle one number)

Not at all…………………………………………………………	1
Less than once a week………………………………………	2
About once a week……………………………………………	3
Several times a week…………………………………………	4
Daily…………………………………………………………………	5

7. Overall, how would you rate your ability to function sexually **during the last 4 weeks**? (Circle one number)

Very poor…………………………………………………………	1
Poor…………………………………………………………………	2
Fair…………………………………………………………………	3
Good…………………………………………………………………	4
Very good…………………………………………………………	5

8. How big a problem **during the last 4 weeks**, if any, has each of the following been for you? (Circle one number on each line)

	No Problem	Very small Problem	Small Problem	Moderate Problem	Big Problem
a. Your level of sexual desire...........	0	1	2	3	4
b. Your ability to have an erection....	0	1	2	3	4
c. Your ability to reach orgasm.........	0	1	2	3	4

9. Overall, how big a problem has your sexual function or lack of sexual function been for you **during the last 4 weeks**?

No problem………………………………………………………	1
Very small problem…………………………………………	2
Small problem…………………………………………………	3
Moderate problem……………………………………………	4
Big problem………………………………………………………	5
