# Supplementary material for: Treatment decision satisfaction and regret after focal HIFU for localized prostate cancer
Source: World J Urol. 2020 Jun 12;39(4):1121–9. doi: 10.1007/s00345-020-03301-0 (PMC8124049; doi:10.1007/s00345-020-03301-0)
Supplement: Supplementary file 2 — Supplementary file2 (PDF 57 kb) [file 345_2020_3301_MOESM2_ESM.pdf]

Title: Treatment decision satisfaction and regret after focal HIFU for localized prostate cancer

Journal: World Journal of Urology

Authors: Niklas Westhoff, Ramona Ernst, Karl Friedrich Kowalewski, Laura Schmidt, Thomas Stefan Worst, Maurice-Stephan Michel, Jost von Hardenberg

Corresponding author:

Niklas Westhoff, M.D.

Department of Urology and Urosurgery, Medical Faculty Mannheim, Heidelberg University, Mannheim, Germany

Theodor-Kutzer-Ufer 1 - 3

68167 Mannheim, Germany

Fon: +49 621 383 8331

Fax: +49 621 383 2184

Email: niklas.westhoff@medma.uni-heidelberg.de

Prostate Cancer-Related Quality of Life Scales

Clark et al., Journal of Clinical Oncology. 2003 Oct 15;21(20):3777-84

Domain	Survey Item
Treatment regret	I wonder if I would have been better off with a different treatment
	I sometimes wonder whether it was really worthwhile being treated at all
	I sometimes feel the treatment I had was the wrong one for me
	If I had to do over, I would choose some other treatment
	I sometimes wish I could change my mind about the kind of treatment I chose for my prostate cancer
Informed decision	I had all the information I needed when a treatment was chosen for my prostate cancer
	My doctors told me the whole story about the effects of treatment
	I knew the right questions to ask my doctor
	I had enough time to make a decision about my treatment
Health worry	My health could take a turn for the worse at any time
	I sometimes worry about dying before my time
	I worry about what my doctor will find next
	I worry that changes in my medical condition will not be detected early
	I am uneasy about the present state of my health
	I live in fear that my PSA will rise
PSA concern	I keep close track of my PSA
	Knowing my PSA level is comforting to me
Outlook	I feel that my cancer has given me a better outlook on life
	I feel that coping with cancer has made me a stronger person

Response Scale for All Items

Not at All	A Little Bit	Somewhat	Quite a Bit	Very Much
1	2	3	4	5
