# Supplementary material for: Treatment decision satisfaction and regret after focal HIFU for localized prostate cancer
Source: World J Urol. 2020 Jun 12;39(4):1121–9. doi: 10.1007/s00345-020-03301-0 (PMC8124049; doi:10.1007/s00345-020-03301-0)

Title: Treatment decision satisfaction and regret after focal HIFU for localized prostate cancer

Journal: World Journal of Urology

Authors: Niklas Westhoff, Ramona Ernst, Karl Friedrich Kowalewski, Laura Schmidt, Thomas Stefan Worst, Maurice-Stephan Michel, Jost von Hardenberg

Corresponding author:

Niklas Westhoff, M.D.

Department of Urology and Urosurgery, Medical Faculty Mannheim, Heidelberg University, Mannheim, Germany

Theodor-Kutzer-Ufer 1 - 3

68167 Mannheim, Germany

Fon: +49 621 383 8331

Fax: +49 621 383 2184

Email: niklas.westhoff@medma.uni-heidelberg.de

ROC curve for the combination of cancer recurrence and general health worry as predictors of regret of focal therapy

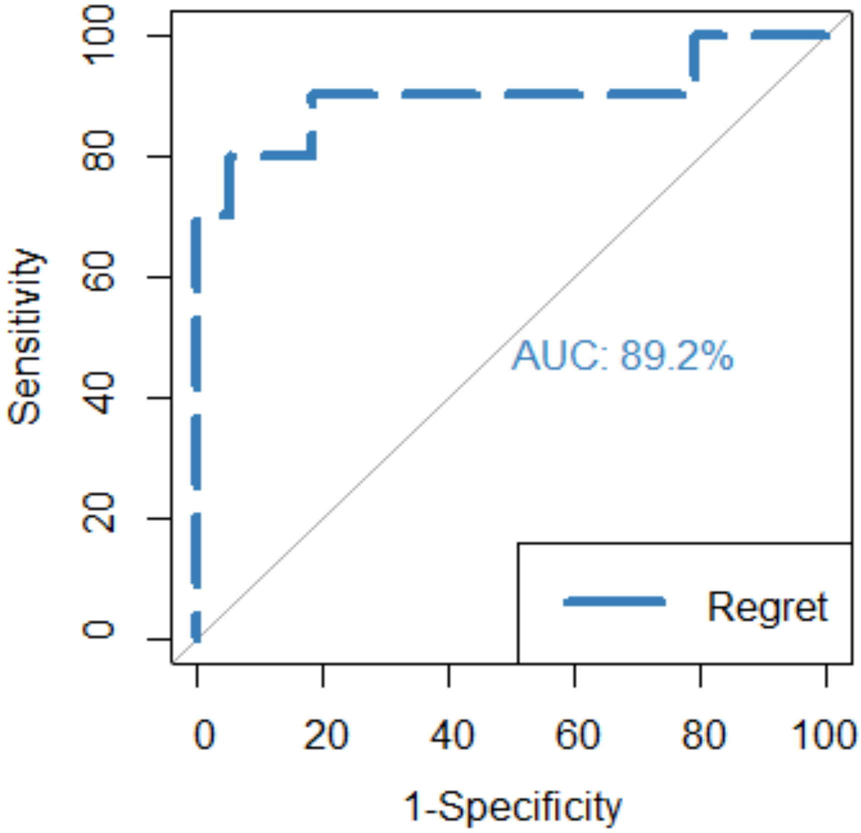

Supplement: Supplementary file 3 — Supplementary file3 (PDF 37 kb) [file 345_2020_3301_MOESM3_ESM.pdf]
